# Trends and variation in issuance of high‐cost narcolepsy drugs by NHS England organisations and regions from 2019 to 2022

**DOI:** 10.1111/jsr.14415

**Published:** 2024-12-08

**Authors:** Frederick van Someren, Milan Wiedemann, Charlotte Warren‐Gash, Martina Sykorova, Hema Mistry, Michelle A. Miller, Guy Leschziner, Ellen Nolte, Aurélien Belot, Ian E. Smith, Tim Quinnell, Sofia H. Eriksson, Helen Strongman

**Affiliations:** ^1^ National Hospital for Neurology and Neurosurgery London UK; ^2^ The Bennett Institute for Applied Data Science, Nuffield Department of Primary Care Health Sciences University of Oxford Oxford UK; ^3^ Faculty of Epidemiology and Population Health London School of Hygiene and Tropical Medicine London UK; ^4^ Warwick Medical School University of Warwick Warwick UK; ^5^ Department of Neurology and Sleep Disorders Centre Guy's and St Thomas' NHS Foundation Trust London UK; ^6^ Faculty of Public Health and Policy London School of Hygiene and Tropical Medicine London UK; ^7^ Respiratory Support and Sleep Centre Royal Papworth Hospital Cambridge UK; ^8^ Department of Clinical and Experimental Epilepsy, UCL Institute of Neurology University College London London UK; ^9^ Narcolepsy UK Cambridgeshire UK

**Keywords:** narcolepsy, pharmacoepidemiology, pitolisant, sodium oxybate, solriamfetol

## Abstract

Clinicians and people with narcolepsy report varied access to higher‐cost narcolepsy treatments in England associated with variations in national and local commissioning. There are no publicly available data quantifying use of these drugs to support policy decisions. We therefore aimed to describe national, regional and local prescribing trends for higher‐cost narcolepsy drugs using new national databases. We used the English prescribing dataset and secondary care medicines data to quantify volumes of high‐cost narcolepsy drugs issued between 01 January 2019 and 31 December 2022. Volumes were converted to World Health Organisation defined daily doses, to estimate the monthly number of defined daily doses of sodium oxybate, pitolisant and solriamfetol issued by each integrated care board and region. We compared national, integrated care board, and regional level issuance of each drug over time. Analysis of almost 6000 primary care prescriptions and 2000 cumulative months of secondary care pharmacy stock data, issued across 41/42 integrated care boards in England, revealed a 49.1% increase in issuance of high‐cost narcolepsy drugs between 2019 and 2022. In 2022, sodium oxybate accounted for 52.66% of issuance, pitolisant 43.09% and solriamfetol 4.25%, with 22.31% of defined daily doses issued in primary care. Three integrated care boards (NHS Southeast London, NHS Cumbria and North‐East, NHS Cheshire and Merseyside) predominate, issuing 56.33% of all defined daily doses. Variations between integrated care boards and regions differ substantially by drug and route of issuance. Our findings describe substantial variation in the use of specialist narcolepsy drugs in England, and highlight the untapped potential of using large, public domain datasets to publicly review higher‐cost drug prescribing.

## 1 INTRODUCTION

Narcolepsy is a chronic neurological disorder characterised by excessive daytime sleepiness (EDS), disrupted sleep–wake cycles, cataplexy, hallucinations and sleep paralysis (Thorpy & Dauvilliers, 2015). The European prevalence of narcolepsy is widely cited as approximately 0.047% (95% confidence interval [CI] 0.016%–0.078%), although estimates vary substantially (Ohayon et al., 2002). A recent estimate using routinely collected National Health Service (NHS) data suggests a diagnosed prevalence of approximately 0.02% of people in England (Strongman et al., 2023). As there is no known cure for narcolepsy, pharmacological treatment and lifestyle management focus on symptom control; despite this, narcolepsy has a substantial lifelong impact on every aspect of patients’ lives (Narcolepsy UK, 2019). Excessive daytime sleepiness has traditionally been managed with low‐cost generic stimulant drugs, including modafinil, dexamphetamine and methylphenidate. In England, these drugs are primarily prescribed by neurologists and respiratory physicians with an interest in sleep, at specialist sleep centres, sometimes supported by shared care agreements with primary care practices. Newer, higher‐cost treatment options include sodium oxybate, pitolisant and solriamfetol. Based on the availability of placebo‐controlled randomised clinical trial evidence, these are recommended as first‐line treatments for certain patients by European guidelines, alongside the more established treatment modafinil (Bassetti et al., 2021). There is no clinical trial evidence for dexamphetamine and methylphenidate in people with narcolepsy, and no evidence comparing the effectiveness of older and newer drugs. In England, prescribing decisions are governed variously by integrated care boards (ICBs; statutory bodies responsible for planning/funding most local NHS services), NHS England and the National Institute for Health and Care Excellence (NICE; Box 1). Clinicians and patients in England have reported varied access to and routes of prescribing of narcolepsy treatments, reflecting evidence limitations and the complexity of commissioning decisions (Narcolepsy UK, 2019; Zeman & Zaiwalla, 2016).

Policy decisions and service planning governing the use of interventions in national health systems relies on real‐world data to characterise routine practice and describe variation in care. In England, this is recognised by NICE's Real‐World framework. Thus far, the absence of publicly‐available data for specialist‐prescribed medications has made it impossible to quantify prescribing and variation in the use of higher‐cost drugs (HCDs; Goldacre & Mackenna, 2020). NICE's solriamfetol technology appraisal therefore relied on clinical experts and unpublished NHS formulary data provided by the company to identify standard care pathways and relevant comparators. The publication of secondary care medicines data (SCMD; via the NHS‐Business Services Authority [NHS‐BSA] since 2020) provides an opportunity to review specialist prescribing in secondary care. This resource complements the English prescribing dataset (EPD), which has been available since 2010 and describes prescribing in primary care.

Using these datasets, this study sought to quantify trends in the use of high‐cost, narcolepsy drugs, and compare levels of prescribing at national, regional and ICB levels.

BOX 1 **Funding and commissioning of higher‐cost narcolepsy drugs in England**

**Table:** 

	Sodium oxybate	Pitolisant	Solriamfetol
Funding	ICBs (adults) Direct commissioning by NHS England (children age 7–19 years with narcolepsy type 1) (NHS England, 2021)	ICBs	ICBs
National review	NHS England (children) Non‐binding Regional Medicines Optimisation Advisory Statement (2019) (Specialist Pharmacy Service, 2019)	NICE evidence review decided not to refer for technology appraisal (NICE 2017) Paediatric Indication selected for technology appraisal (NICE 2024)	NICE approval for treating EDS caused by narcolepsy for those for whom existing low‐cost generics have not worked or are not suitable (NICE 2022)
Decision making	Regional decision making in the absence of national guidance	Regional decision making in the absence of national guidance	Commissioning mandated by ICBs in line with NICE guidance (NICE TA758, 2022)
Cost	£540–£1080 per patient per month (British National Formulary, 2024a)	£310–£620 per patient per month (NICE, 2024a)	£192 to £271 per patient per month (British National Formulary, 2024a)

EDS, excessive daytime sleepiness; ICB, integrated care board; NHS, National Health Service; NICE, National Institute for Health and Care Excellence.

## 2 METHODS

### 2.1 Study design

This retrospective, observational, analysis of HCDs used in narcolepsy was conducted using two publicly available datasets provided by the NHS‐BSA.

Prescribing and Pharmacy stock issuance data were collated from: (1) the EPD; and (2) the SCMD, covering the period January 2019–December 2022. These datasets provide monthly data related to volumes of medicines and pharmaceutical stock issued in primary and secondary care, respectively, for all general practitioners and secondary care trusts in NHS England over a given period.

Total volumes of virtual medicinal product (VMP) issued per medication by each GP practice or hospital trust are recorded, alongside relevant treatment meta‐data. Detailed information about all data sources is provided in Appendix S1 in Data S1.

### 2.2 Procedures

Solriamfetol, pitolisant and sodium oxybate prescription and stock issuance data were identified in the EPD and SCMD by their “BNF Chemical Product” (i.e. the active ingredient of the medication) and VMP SNOMED “concept” codes (Appendix S1 in Data S1), respectively. Volumes of VMP issued each month per GP practice or secondary care trust were collated and combined with meta‐data from the NHS Dictionary of Medicines and devices describing treatment‐specific “VMP quantity” (i.e. the aliquots in which medications are issued by pharmacy) and strength, to calculate a total amount of milligrams of active ingredient issued each month (Box 2).

BOX 2 **Conversion of VMP volumes to DDDs, calculated per month per GP practice/trust**

$$\text{Volume DDDs}=\frac{\left(\mathrm{VMP}\;\text{Quantity}÷\text{UDFS}\right)\times \left(\text{Strength of Single Drug Unit}\right)}{\mathrm{WHO}\;\mathrm{DDD}}$$

VMP quantity = total volume of medication issued per unit time by a GP practice/trust; UDFS = unit dose form size (e.g. pitolisant 18 mg tablets have a UDFS of 1 as each tablet is 18 mg); “Strength of single drug unit” as defined by SNOMED CT (e.g. a single tablet of 18 mg pitolisant has a strength of 18 mg); adjustments are required for medications with differing unit strength and DDD (e.g. sodium oxybate single unit strength = 500 mg, whilst DDD = 7.5 g).

DDD, defined daily dose; VMP, virtual medicinal product; WHO, World Health Organisation.

Total quantities of active ingredients were converted to volumes of World Health Organisation (WHO) defined daily doses (DDD), facilitating direct comparison of treatments. There is no WHO DDD for solriamfetol; solriamfetol was therefore assigned a DDD of 150 mg based on the treatment dose required to meet primary endpoints observed in phase 2 and 3 trials (Ruoff et al., 2016; Thorpy et al., 2019), and the maximum of two doses recommended in the marketing authorisation (British National Formulary, 2024b).

Finally, NHS trusts were mapped to ICBs and geographical regions by combining unique Organisation Data Service (ODS) codes identifying each trust in the SCMD data to the NHS Digital General Practice Mapping File and NHS Digital “etr” data. Fifteen ODS codes in SCMD were corrected to their current codes as per the “etr” dataset. EPD prescriptions not associated with an identifiable practice or ICB code were omitted (2.35% of sodium oxybate and 2.46% of pitolisant prescriptions, respectively). One month of SCMD stock data from one trust (March 2020, Northern Care Alliance NHS Foundation Trust) was excluded as a substantial negative stock issuance was reported (equivalent to ~ −20,000 DDDs) and considered to be a data error.

### 2.3 Descriptive analysis

We analysed total monthly volumes of DDDs issued in primary and secondary care for sodium oxybate, pitolisant and solriamfetol by each ICB across NHS England between 2019 and 2022. We described the total DDDs of each drug issued nationally (number and proportion), over time (number as a 3‐month rolling average and proportion in 2019 and 2022), by ICB (proportion in 2019, 2022 and over the full time period) and by geographic region (number across full time period).

### 2.4 Code sharing

All data management processes and analyses were completed using R Version 4.4.0 through *RStudio*. All code‐lists, data and analysis codes are available in our online repository (https://tinyurl.com/25ud9sbe). Key definitions are provided in Appendix S1 in Data S1, alongside code‐lists and key dm + d data for solriamfetol, pitolisant and sodium oxybate.

## 3 RESULTS

### 3.1 National trends

**FIGURE 1:**
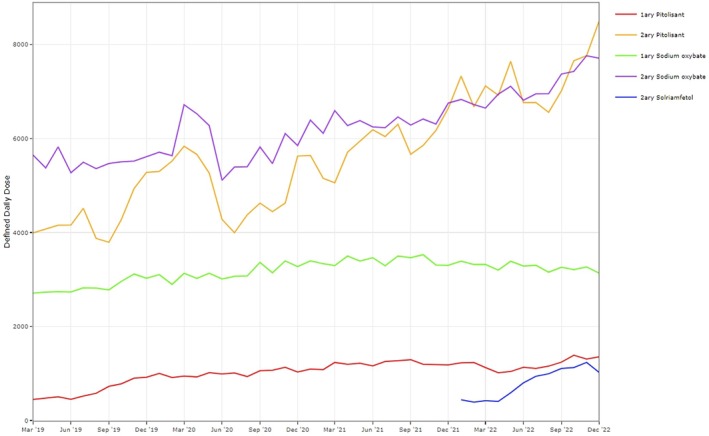
Three‐month rolling average of combined issuance rates (defined daily doses [DDDs] issued) for pitolisant, sodium oxybate and solriamfetol from primary care services and all secondary care trusts in England during the period January 2019 to December 2022. Solriamfetol was first issued in December 2021.

National trends in issuance of HCDs used to treat narcolepsy are described in Table 1 and Figure 1. Between January 2019 and December 2022, there were 778,415 DDDs of HCDs issued (i.e. supplied to patients through community or hospital pharmacies) across NHS England, consisting of 449,851 DDDs of sodium oxybate (57.79%), 317,753 DDDs of pitolisant (40.82%) and 10,811 DDDs of solriamfetol (1.39%). The volume of DDDs of HCDs issued was higher in secondary care (582,282 DDDs, 74.80%) than in primary care (196,133 DDDs, 25.20%). Yearly issuance of HCDs increased by 49.1% over the 4‐year study period. This was driven, largely, by increased issuance of pitolisant and, to a lesser degree, sodium oxybate and solriamfetol in secondary care. By 2022, the proportion of DDDs of pitolisant issued in secondary care (37.3%) was slightly higher than sodium oxybate (36.2%); in primary care, the proportion of DDDs of sodium oxybate (16.5%) was greater than pitolisant (5.8%). The remaining 4.3% of DDDs issued were solriamfetol in secondary care.

**TABLE 1:** Summary table of volumes of DDDs of each HCD issued in primary and secondary care, and the total number of ICBs issuing each medication between January 2019 and December 2022.

	Total DDDs (2019–2022)		Total DDDs (2019)		Total DDDs (2022)		STPs/ICBs issuing (out of 42)	
	DDDs	% DDDs	DDDs	% DDDs	DDDs	% DDDs	*n*	%
Sodium oxybate								
Primary care	150,012	19.27	33,756	21.27	39,012	16.48	36	85.71
Secondary care	299,839	38.52	66,017	41.59	85,645	36.18	28	66.67
Total	449,851	57.79	99,773	62.86	124,657	52.66	40	95.24
Pitolisant								
Primary care	46,121	5.92	7277	4.58	13,801	5.83	21	50.00
Secondary care	271,633	34.90	51,669	32.55	88,188	37.26	17	40.48
Total	317,753	40.82	58,945	37.14	101,989	43.09	26	61.90
Solriamfetol								
Primary care	0	0.00	0.00	NA	0.00	NA	0	0.00
Secondary care	10,811	1.39	0.00	NA	10,055	4.25	17	40.48
Total	10,811	1.39	0.00	NA	10,055	4.25	17	40.48
All drugs combined								
Primary care	196,133	25.20	41,033	25.85	52,813	22.31	38	90.48
Secondary care	582,282	74.80	117,686	74.15	183,888	77.69	29	69.05
Total	778,415	100.00	158,719	100.00	236,701	100.00	41	97.62

The percentage of DDDs indicates the percentage total for each treatment in primary and secondary care, and the combined total, in the specified time period.

DDDs, defined daily doses; ICB, integrated care board; STP, Sustainability and Transformation Plan.

#### 3.1.1 Trends by ICB

**FIGURE 2:**
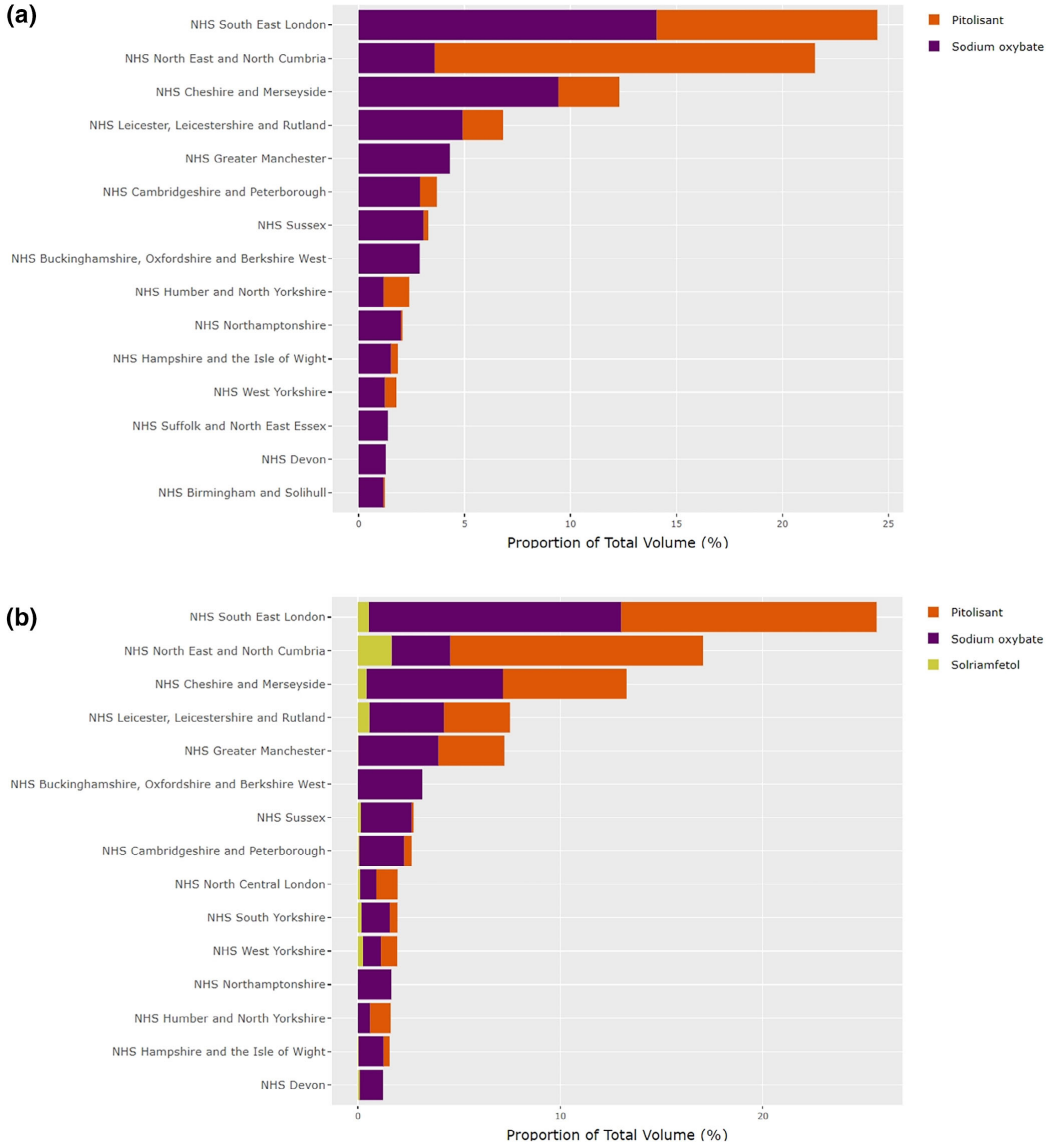
Proportion of defined daily doses (DDDs) issued per integrated care board (ICB) in (a) 2019 and (b) 2022 as a percentage of total volume issued within National Health Service (NHS) England in that year. The figures are restricted to the 15 ICBs that issued the highest volumes of higher‐cost drugs (HCDs) for narcolepsy.

Across the study period, 41/42 ICBs issued HCDs for narcolepsy, 38 and 29 of which issued HCDs in primary care and secondary care, respectively (Table 1). Overall, three ICBs (NHS South East London, NHS North East and North Cumbria, NHS Cheshire and Merseyside) accounted for 58.3% and 55.9% of HCD issuance in 2019 and 2022, respectively (Figure 2; Table S1). These three ICBs accounted for 71.76% of DDDs issued in secondary care across the study period (Table S2), and were early adopters of pitolisant (Figure 2). In primary care, NHS Leicester, Leicestershire and Rutland; NHS Cambridgeshire and Peterborough; and NHS Northamptonshire accounted for 41.39% of the total DDDs issued (Table S3).

Relative proportions of HCDs issued vary between ICBs. For example, in 2022, NHS South East London had issued 25.62% of the total national DDDs, of which 48.62% were sodium oxybate, 49.26% pitolisant and 2.11% solriamfetol. In contrast, NHS North East and Cumbria had issued 17.05% of the total national DDDs, of which 16.95% of were sodium oxybate, 73.26% pitolisant and 9.79% solriamfetol (Figure 2b; Table S1b).

#### 3.1.2 Geographical variation

**FIGURE 3:**
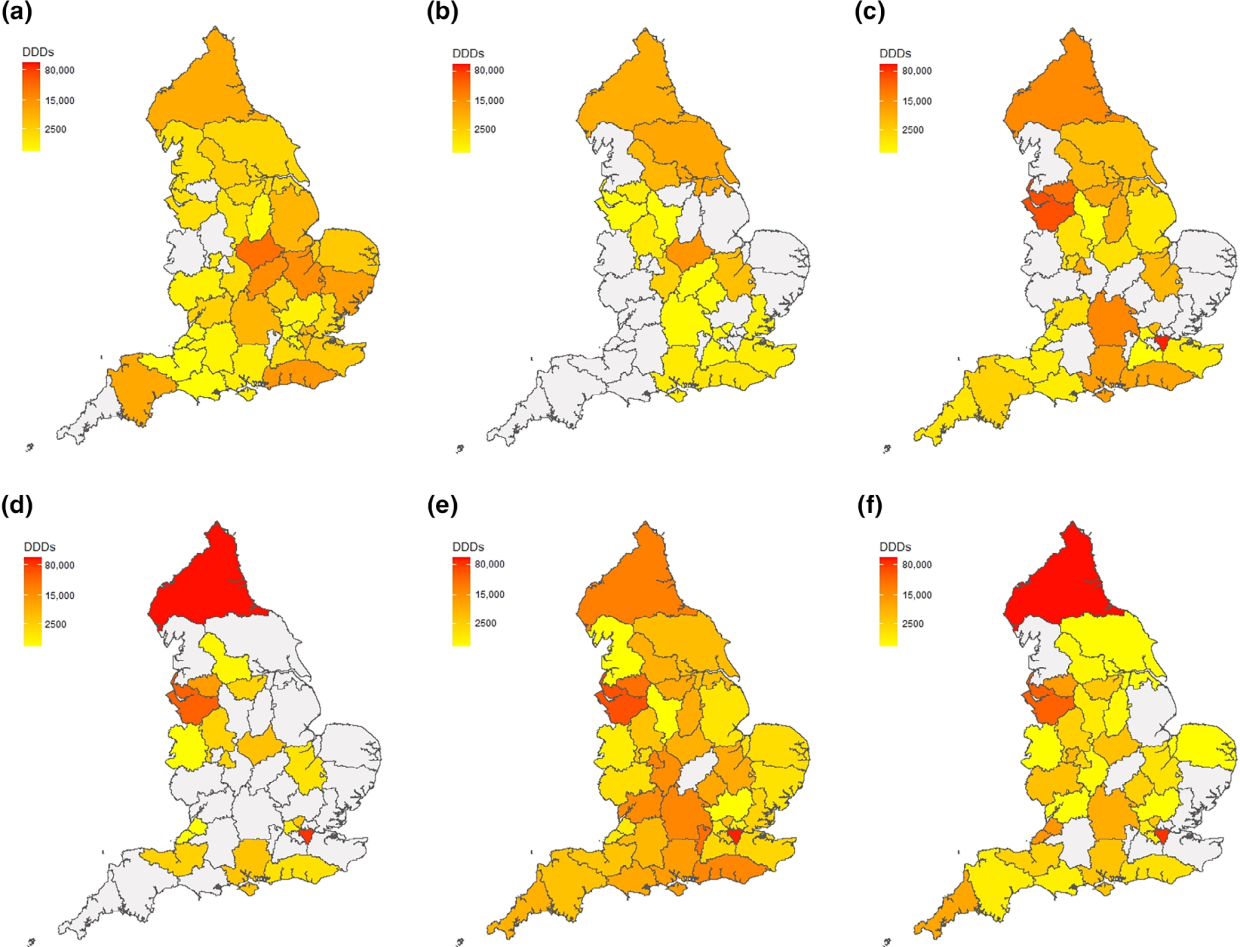
Volumes of defined daily doses (DDDs) of higher‐cost drugs (HCDs) used to treat narcolepsy issued per integrated care board (ICB) in primary care (a: sodium oxybate; b: pitolisant), secondary care (c: sodium oxybate; d: pitolisant), and combined volumes issued in both primary and secondary care (e, f) between January 2019 and December 2022. Geographic data for sustainability and transformation plan (STP)/ICB was obtained from the UK Office for National Statistics (ONS) Open Geography portal (Office for National Statistics‐Open Geography Portal, 2023).

**FIGURE 4:**
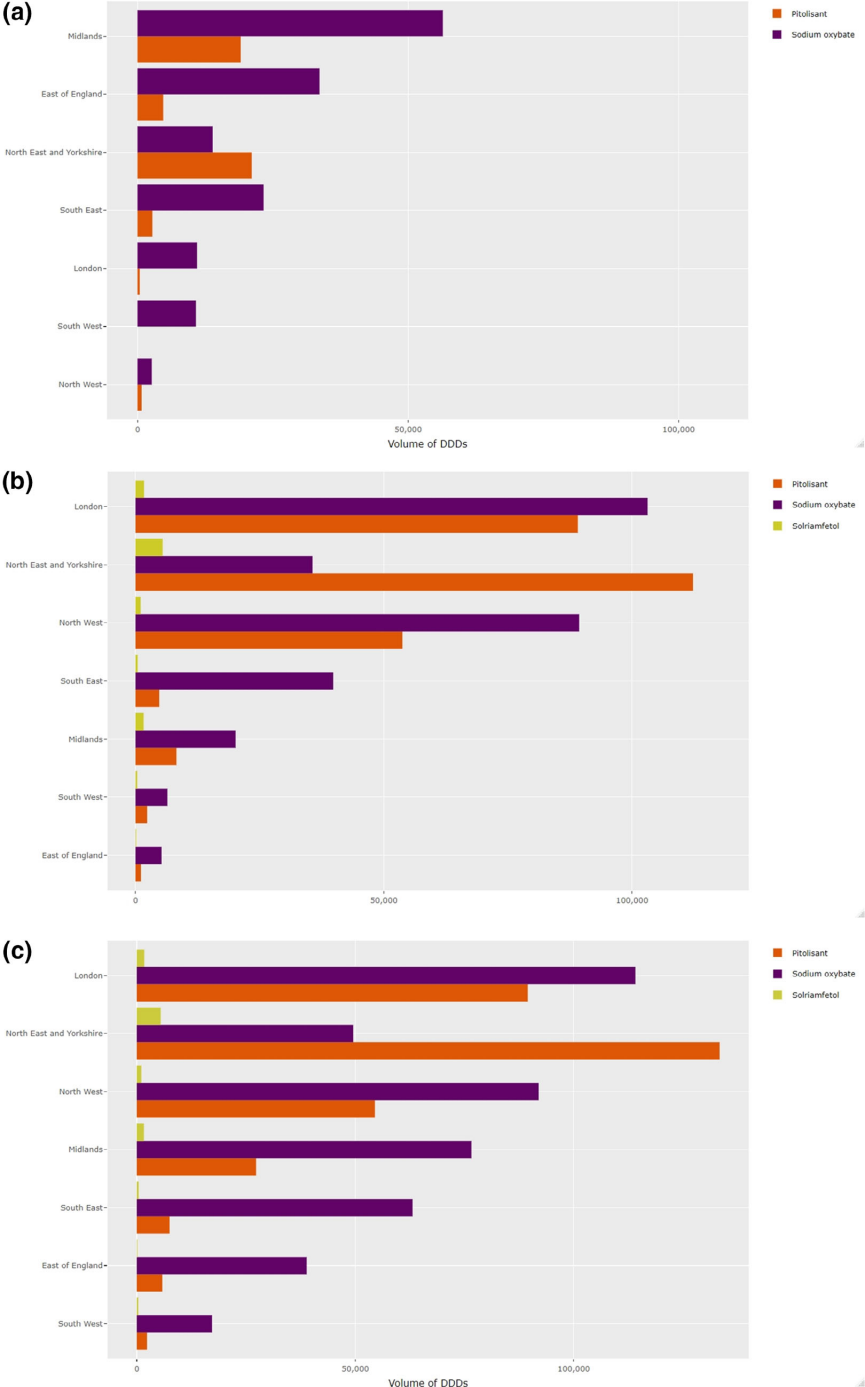
Total volumes of defined daily doses (DDDs) of higher‐cost drugs (HCDs) issued in different geographical regions of National Health Service (NHS) England between 2019 and 2022 in (a) primary care, (b) secondary care, and (c) combined volumes in primary and secondary care.

Figure 3(a–d) describes geographical variation in issuance of each HCD in primary and secondary care for all ICBs across the study period. Regional patterns that are evident in Figure 3 are summarised in Figure 4 and Table S4. In line with trends by ICBs described above, issuance of HCDs was highest in London, the North East & Yorkshire and the North West. Similarly high volumes of primary care issuance of sodium oxybate, and to a lesser extent pitolisant, in three ICBs in the Midlands and East of England accounted for a substantial minority of the overall volumes issued in these regions (Tables S2 and S3). In the South East, issuance of sodium oxybate was moderate and issuance of pitolisant was low in both primary and secondary care. In the South West, issuance of all HCDs in both primary and secondary care was low.

**FIGURE 5:**
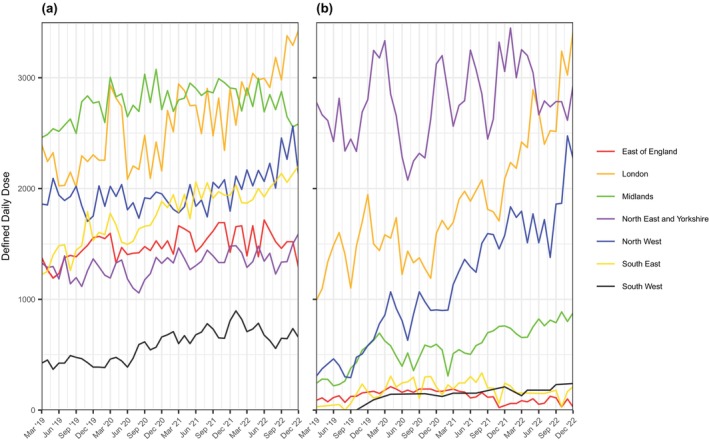
Three‐monthly rolling averages of defined daily doses (DDDs) of (a) sodium oxybate and (b) pitolisant issued across the National Health Service (NHS) England regions between 2019 and 2022.

These findings are reflected in regional trends observed over time, with average volumes of DDDs issued across all regions (except East of England) increasing over the observation period (Figure 5). Whilst the absolute increase of sodium oxybate DDDs issued is driven by the 40.16% increase of DDDs issued in London, there are large increases in pitolisant issuance across several regions: in London (99.10%), Midlands (121.57%) and the North West (386.04%).

## 4 DISCUSSION

To our knowledge, this study is the first to describe prescribing trends of HCDs used to treat narcolepsy in England at national, regional and ICB levels. We demonstrate that at the national level, volumes of issuance of sodium oxybate, pitolisant and solriamfetol in secondary care increased between January 2019 and December 2022, but prescribing was not evenly distributed across ICBs and regions. Notably, three ICBs (NHS Southeast London, NHS Cumbria and North East, NHS Cheshire and Merseyside) accounted for over half of all HCD DDDs issued in 2022 (56.11%). A quarter of DDDs in 2022 were issued in primary care. There was substantial variation in prescribing patterns for sodium oxybate compared with pitolisant, and volumes issued in primary versus secondary care.

### 4.1 Strengths and limitations

The strength of this study lies in the large repository of prescribing and pharmacy stock data that are now provided by the EPD and the SCMD. These databases included all primary care and narcolepsy treatment centres in NHS England over an extended 4‐year observation period. This enabled clear delineation of regional variations in issuance of higher‐cost treatments for narcolepsy, and reiterates the strength of utilising SCMD in examining national prescribing trends and habits.

However, there are limitations to the use of the EPD and SCMD. In both datasets there are potential discrepancies between reported treatment issuance and usage. In the EPD, prescriptions issued may not correlate with treatment usage due to patient factors (e.g. non‐adherence, inappropriate use, etc.), whilst SCMD reports pharmaceutical stock (as opposed to issuance of prescriptions), resulting in several data anomalies, such as high monthly variations in reported values and pharmacies issuing negative monthly values of stock. These negative (or null) values may be interpreted in several ways (Okoli et al., 2021). In the context of this study, one pharmacy reported such a negative stock issuance so large it was judged to be erroneous. Additionally, three secondary care trusts only report partial datasets in this observation period, either due to technical limitations in data acquisition (University College London Hospital [UCLH] NHS Foundation Trust & Great Ormond Street Hospital [GOSH] NHS Foundation Trusts) or due to non‐disclosure agreements (Royal Brompton and Harefield NHS Foundation Trust; NHS Business Service Authority, 2021). Consequently, no data were available from UCLH for April 2019–October 2020 or April 2021 to December 2022, from GOSH over the entire data period, or the Royal Brompton in Jan 2019–Dec 2019 or May 2020–Dec 2022. This limited the interpretation of results from the ICB “NHS North Central London”. A small percentage of primary care prescriptions were not included due to absent or inaccurate GP practice codes within the EPD data. These and other unknown data discrepancies cause unquantifiable measurement error.

The EPD and SCMD are aggregated data repositories, which means that it was not possible to identify the rationale behind the issuance of each individual treatment. The treatments considered in this study are only licenced for narcolepsy, enabling meaningful assessment of their usage; however, some DDDs may have been issued, off licence, for other indications, for example, idiopathic hypersomnia. It was not possible to review other, primarily first‐line, narcolepsy treatments (such as modafinil, Lis/Dex‐amphetamine and methylphenidate), which have other medical indications. This restricts comparison of the issuance of these treatments with the HCDs assessed in this study. As narcolepsy treatments are often provided by tertiary centres without a specified catchment area, the denominator population for each geographic area is unknown. In the absence of individual patient‐related data, it was not possible to determine where patients have come from to receive treatment, the number of patients receiving the treatment, or to calculate associated prescribing rates or reasons for variation in care.

Finally, as solriamfetol was assigned a DDD (as there is no formal WHO‐DDD), this may result in the over‐ or underestimation of the true volume of solriamfetol DDDs distributed.

### 4.2 Implications for policy and practice

A 2021/2022 NICE Technology Appraisal consultation for the prescribing of solriamfetol for treating EDS caused by narcolepsy revealed a paucity of reliable publicly available evidence describing the national uptake of individual high‐cost narcolepsy drugs, let alone their regional distribution (NICE, 2021).

Moreover, there is limited evidence regarding prescribing volumes, and variation in access to HCDs extends to other neurological conditions; this has been based on rare and expensive national audits (Pinho‐Gomes et al., 2022) and small, localised studies (Roddam et al., 2019).

Our findings point to unequal geographic access to specialist narcolepsy drugs in England that varies substantially by drug substance. This is supported by a 2012 study describing wide variation in the number of sleep centres per Clinical Commissioning Group (CCG; now ICB) and in the diagnostic facilities available (Steier et al., 2014). While there are regions with lower levels of prescribing despite the existence of specialist sleep services (e.g. North Bristol Trust in the South West), it is probable that some of the ICBs that did not issue high‐cost narcolepsy drugs still do not have dedicated sleep services that have the facilities and expertise to diagnose and treat narcolepsy. Patients in these areas will need to travel to receive care often far from home, limiting access and potentially worsening outcomes (Kelly et al., 2016; Steier et al., 2014). These disparities may be reduced by facilitating prescribing by primary care practices; it is notable that the two ICBs for sodium oxybate issuance in primary care (NHS Leicester, Leicestershire and Rutland, and NHS Cambridgeshire and Peterborough) have established policies to facilitate on‐going community prescribing and funding (Cambridge and Peterborough Integrated Care System, 2022; Leicester Leicestershire and Rutland Area Prescribing Committee, 2015). Difficulties in obtaining referrals may further increase inequalities.

Historically, HCDs for narcolepsy treatment have not been readily accessible for most patients with the disorder, and required individual funding requests to the local CCGs, now ICBs (Sanghvi et al., 2020; Zeman & Zaiwalla, 2016). Such applications require clinicians to show that the individual patient's clinical circumstances are exceptional, and consequently these applications are often declined. As sodium oxybate has not been reviewed by NICE following its launch in 2006, the Regional Medicines Optimisation Committee (RMOC; Midlands and East), which reviews prescribing and commissioning to assist the decision‐making process and improve consistency, issued guidance for sodium oxybate prescribing in October 2019 (Specialist Pharmacy Service, 2019). In 2017, NICE considered referral of pitolisant for a technology appraisal inappropriate (NICE, 2017), although the paediatric indication has more recently been accepted for review (NICE, 2024). In contrast, NICE reviewed and recommended solriamfetol for treatment of narcolepsy with and without cataplexy, following the exclusion of two generic drugs, in a Technology Appraisal published in 2022 (NICE, 2022). While issuing national and regional guidelines may reduce prescribing variation over time, their impact is not evident in the current study either in terms of volume of prescribing or route of issuance. Further research is needed to fully understand the role of national policies, local commissioning decisions, and prescriber preference in explaining variability in issuance of high‐cost narcolepsy drugs. Robust evidence comparing the effectiveness and safety of drugs for EDS in people with narcolepsy is also needed (Bassetti et al., 2021).

Our analysis of EPD and SCMD, together with previous research studying the use of biological medicines for severe asthma (Rowan & MacKenna, 2020), demonstrates how large, publicly available datasets can be used to quantify issuance of HCDs in England, and describe regional and hospital level variance. Further research is needed comparing the proportion of people diagnosed with narcolepsy living in each region who are prescribed high‐cost narcolepsy drugs, where they receive care, and analysing access to care inequalities by a wider range of area and person‐based characteristics. This would require national person‐level HCD prescribing data that includes demographic and wider characteristics or population‐based collection of data from specialist sleep centres about people with narcolepsy. OpenSAFELY demonstrated that it is possible to collect national person‐level HCD prescribing data during the Covid‐19 pandemic (MacKenna et al., 2021), and facilitates analyses that link person‐level prescription data with other health and demographic datasets (OpenSAFELY Collaborative et al., 2021).

While SCMD data are publicly available, and code used to manage and analyse data for both studies has been published, this dataset does not appear to have been widely used since it was first published in January 2019. To maximise use of these data, they need to be presented in a form that is available to a wider group of people. The benefits of such an approach has been demonstrated by the OpenPrescribing platform, which enables exploration of primary care prescribing in the UK, benefiting patient care and safety.

## 5 CONCLUSION

Across the 4 year observation period, we have described a national increase in issuance of higher‐cost treatments used to manage narcolepsy. There is substantial regional and local variation in issuance of HCDs in both primary and secondary care, pointing to the absence of an agreed standard of care and disparities in access to specialist narcolepsy treatments. We have demonstrated the potential of public‐domain datasets such as EPD and SCMD to inform policy decisions and service planning related to the use of higher‐cost treatments for specialist conditions, and discussed the need for patient‐level datasets to allow for more comprehensive analyses.

## AUTHOR CONTRIBUTIONS

**Frederick van Someren:** Investigation; writing – original draft; methodology; writing – review and editing; visualization; formal analysis; data curation. **Milan Wiedemann:** Methodology; data curation; software; writing – review and editing. **Charlotte Warren‐Gash:** Writing – review and editing. **Martina Sykorova:** Writing – review and editing. **Hema Mistry:** Writing – review and editing. **Michelle A. Miller:** Writing – review and editing. **Guy Leschziner:** Writing – review and editing. **Ellen Nolte:** Writing – review and editing. **Aurélien Belot:** Writing – review and editing. **Ian E. Smith:** Writing – review and editing. **Tim Quinnell:** Writing – review and editing. **Sofia H. Eriksson:** Writing – review and editing; writing – original draft. **Helen Strongman:** Writing – review and editing; writing – original draft; conceptualization; methodology; visualization; formal analysis; project administration.

## CONFLICT OF INTEREST STATEMENT

FvS, GL, AB, IES and EN have no conflicts of interest to declare. HS is a voluntary Director and Trustee of Narcolepsy UK, a patient‐led charity, and served as a patient expert for the NICE technology appraisal (TA758) “Solriamfetol for treating excessive daytime sleepiness caused by narcolepsy”. MAM receives royalties from Oxford University Press for two textbooks. SHE is CI on the NIHR FOUND Trial (NIHR203393). SHE is an executive member of the British Sleep Society. SHE has received honoraria for educational activities from Eisai, Fidia, Lincoln and UCB pharma. SHE served as expert advisor for the NICE technology appraisal (TA758) “Solriamfetol for treating excessive daytime sleepiness caused by narcolepsy”. CWG reports research grants from Wellcome and Open Philanthropy.

## Supporting information


**DATA S1.** Supporting Information.

## Data Availability

All data utilised are provided via the URL within the body of the text (https://tinyurl.com/25ud9sbe). Raw data are available directly from the NHS‐BSA (English Prescribing Dataset: https://www.nhsbsa.nhs.uk/prescription‐data/prescribing‐data/english‐prescribing‐data‐epd; Secondary Care Medicines Database: https://opendata.nhsbsa.net/dataset/secondary-care-medicines-data-indicative-price). Study protocols and analysis codes are provided within the GitHub Repository linked above.
