# Supplementary material for: Trends and variation in issuance of high‐cost narcolepsy drugs by NHS England organisations and regions from 2019 to 2022
Source: J Sleep Res. 2024 Dec 8;34(4):e14415. doi: 10.1111/jsr.14415 (PMC12215291; doi:10.1111/jsr.14415)
Supplement: Supplementary file 1 — DATA S1. Supporting Information. [file JSR-34-e14415-s001.docx]

Supplementary Appendix

**Tables**

Table S1 (a) Data for Figure 2 (a) demonstrating the proportion (%) of DDDs of HCDs issued per ICB in 2019, as percentage of the national total, in primary and secondary care combined.

| (A) | Sodium Oxybate | Pitolisant | Combined |
| --- | --- | --- | --- |
| NHS South East London | 14.06 | 10.42 | 24.48 |
| NHS North East and North Cumbria | 3.59 | 17.95 | 21.54 |
| NHS Cheshire and Merseyside | 9.43 | 2.88 | 12.31 |
| NHS Leicester, Leicestershire and Rutland | 4.90 | 1.91 | 6.82 |
| NHS Greater Manchester | 4.31 | 0.00 | 4.31 |
| NHS Cambridgeshire and Peterborough | 2.90 | 0.80 | 3.69 |
| NHS Sussex | 3.06 | 0.23 | 3.29 |
| NHS Buckinghamshire, Oxfordshire and Berkshire West | 2.88 | 0.00 | 2.88 |
| NHS Humber and North Yorkshire | 1.18 | 1.21 | 2.39 |
| NHS Northamptonshire | 2.00 | 0.07 | 2.07 |
| NHS Hampshire and the Isle of Wight | 1.52 | 0.33 | 1.85 |
| NHS West Yorkshire | 1.24 | 0.55 | 1.78 |
| NHS Suffolk and North East Essex | 1.38 | 0.00 | 1.38 |
| NHS Devon | 1.28 | 0.00 | 1.28 |
| NHS Birmingham and Solihull | 1.17 | 0.07 | 1.24 |
| NHS Nottingham and Nottinghamshire | 1.21 | 0.00 | 1.21 |
| NHS Lincolnshire | 1.16 | 0.00 | 1.16 |
| NHS South Yorkshire | 0.86 | 0.01 | 0.87 |
| All other ICBs (18) | 4.59 | 0.84 | 5.42 |
| Total | 62.73 | 37.27 | 100 |

(b) Data for Figure 2 (b) - demonstrating the proportion of DDDs of HCDs issued per ICB in 2022, as a percentage of the national total, in primary and secondary care combined.

|  | Sodium Oxybate | Pitolisant | Solriamfetol | Combined DDDs |
| --- | --- | --- | --- | --- |
| NHS South East London | 12.46 | 12.62 | 0.54 | 25.62 |
| NHS North East and North Cumbria | 2.89 | 12.49 | 1.67 | 17.05 |
| NHS Cheshire and Merseyside | 6.74 | 6.11 | 0.42 | 13.27 |
| NHS Leicester, Leicestershire and Rutland | 3.68 | 3.26 | 0.57 | 7.51 |
| NHS Greater Manchester | 3.96 | 3.26 | 0.02 | 7.23 |
| NHS Buckinghamshire, Oxfordshire and Berkshire West | 3.18 | 0.00 | 0.00 | 3.18 |
| NHS Sussex | 2.51 | 0.10 | 0.15 | 2.75 |
| NHS Cambridgeshire and Peterborough | 2.22 | 0.37 | 0.06 | 2.66 |
| NHS North Central London | 0.80 | 1.05 | 0.11 | 1.96 |
| NHS South Yorkshire | 1.40 | 0.38 | 0.18 | 1.96 |
| NHS West Yorkshire | 0.88 | 0.81 | 0.25 | 1.94 |
| NHS Northamptonshire | 1.65 | 0.00 | 0.00 | 1.65 |
| NHS Humber and North Yorkshire | 0.60 | 1.02 | 0.00 | 1.62 |
| NHS Hampshire and the Isle of Wight | 1.23 | 0.30 | 0.04 | 1.57 |
| NHS Devon | 1.16 | 0.00 | 0.08 | 1.24 |
| NHS Suffolk and North East Essex | 1.14 | 0.00 | 0.00 | 1.14 |
| NHS Birmingham and Solihull | 0.79 | 0.10 | 0.02 | 0.91 |
| NHS North East London | 0.73 | 0.00 | 0.00 | 0.73 |
| All other ICBs (20) | 4.48 | 1.40 | 0.13 | 6.01 |
|  | 52.50 | 43.27 | 4.23 | 100.00 |

Supplementary Table 2: Summary totals of DDDS issued per ICB in Secondary Care from January 2019 to December 2022, with proportions of the total number of secondary care DDDs issued per ICB.

| ICB | Sodium Oxybate (DDDs) | Proportion (%) | Pitolisant (DDDs) | Proportion (%) | Solriamfetol (DDDs) | Proportion (%) | Combined  (DDDs) | Proportion (%) |
| --- | --- | --- | --- | --- | --- | --- | --- | --- |
| **NHS South East London** | 98136 | 16.85 | 85087.5 | 14.61 | 1456 | 0.25 | 184679.5 | 31.72 |
| **NHS North East and North Cumbria** | 17687.6 | 3.04 | 109765 | 18.85 | 4347 | 0.75 | 131799.6 | 22.64 |
| **NHS Cheshire and Merseyside** | 57768 | 9.92 | 42547.5 | 7.31 | 1008 | 0.17 | 101323.5 | 17.40 |
| **NHS Greater Manchester** | 31596 | 5.43 | 11206 | 1.92 | 42 | 0.01 | 42844 | 7.36 |
| **NHS Buckinghamshire,**  **Oxfordshire and Berkshire West** | 18948 | 3.25 | 0 | 0.00 | 0 | 0.00 | 18948 | 3.25 |
| **NHS Hampshire and the Isle of Wight** | 10956 | 1.88 | 3596 | 0.62 | 84 | 0.01 | 14636 | 2.51 |
| **NHS South Yorkshire** | 8347.2 | 1.43 | 2280 | 0.39 | 420 | 0.07 | 11047.2 | 1.90 |
| **NHS Sussex** | 9192 | 1.58 | 1182.75 | 0.20 | 350 | 0.06 | 10724.75 | 1.84 |
| **NHS Birmingham and Solihull** | 8364 | 1.44 | 1980 | 0.34 | 42 | 0.01 | 10386 | 1.78 |
| **NHS North Central London** | 4848 | 0.83 | 3285 | 0.56 | 266 | 0.05 | 8399 | 1.44 |
| **NHS Nottingham and Nottinghamshire** | 6938.4 | 1.19 | 0 | 0.00 | 140 | 0.02 | 7078.4 | 1.22 |
| **NHS Leicester, Leicestershire and Rutland** | 1188 | 0.20 | 3980 | 0.68 | 1428 | 0.25 | 6596 | 1.13 |
| **NHS Cambridgeshire and Peterborough** | 5256 | 0.90 | 1106.75 | 0.19 | 140 | 0.02 | 6502.75 | 1.12 |
| **NHS West Yorkshire** | 5268 | 0.90 | 247.5 | 0.04 | 714 | 0.12 | 6229.5 | 1.07 |
| **NHS Humber and North Yorkshire** | 4352 | 0.75 | 0 | 0.00 | 0 | 0.00 | 4352 | 0.75 |
| **NHS Staffordshire and Stoke-on-Trent** | 1248 | 0.21 | 2251 | 0.39 | 28 | 0.00 | 3527 | 0.61 |
| **NHS Somerset** | 661.3333 | 0.11 | 2326.5 | 0.40 | 0 | 0.00 | 2987.833 | 0.51 |
| **NHS Devon** | 2268 | 0.39 | 0 | 0.00 | 196 | 0.03 | 2464 | 0.42 |
| **NHS Gloucestershire** | 1392 | 0.24 | 0 | 0.00 | 126 | 0.02 | 1518 | 0.26 |
| **NHS Black Country** | 1512 | 0.26 | 0 | 0.00 | 0 | 0.00 | 1512 | 0.26 |
| **NHS Cornwall and the Isles of Scilly** | 996 | 0.17 | 0 | 0.00 | 0 | 0.00 | 996 | 0.17 |
| **NHS Lincolnshire** | 900 | 0.15 | 0 | 0.00 | 0 | 0.00 | 900 | 0.15 |
| **NHS North West London** | 156 | 0.03 | 723.5 | 0.12 | 0 | 0.00 | 879.5 | 0.15 |
| **NHS Kent and Medway** | 720 | 0.12 | 0 | 0.00 | 0 | 0.00 | 720 | 0.12 |
| **NHS Dorset** | 612 | 0.11 | 0 | 0.00 | 0 | 0.00 | 612 | 0.11 |
| **NHS Bristol, North Somerset**  **and South Gloucestershire** | 504 | 0.09 | 30 | 0.01 | 24 | 0.00 | 558 | 0.10 |
| **NHS Shropshire, Telford and Wrekin** | 0 | 0.00 | 37.5 | 0.01 | 0 | 0.00 | 37.5 | 0.01 |
| **NHS Derby and Derbyshire** | 12 | 0.00 | 0 | 0.00 | 0 | 0.00 | 12 | 0.00 |
| **NHS Surrey Heartlands** | 12 | 0.00 | 0 | 0.00 | 0 | 0.00 | 12 | 0.00 |
| **National Totals** | 299838.5 | 51.49 | 271632.5 | 46.65 | 10811 | 1.86 | 582282 |  |

Supplementary Table 3: Summary totals of DDDs issued per ICB in primary care from January 2019 to December 2022, with proportions of the total number of primary care DDDs issued per drug per ICB.

| ICB | Sodium Oxybate DDDs | Proportion (%) | Pitolisant | Proportion (%) | Combined | Proportion |
| --- | --- | --- | --- | --- | --- | --- |
| NHS Leicester, Leicestershire and Rutland | 31428 | 15.67 | 15849 | 7.90 | 47277 | 23.57 |
| NHS Cambridgeshire and Peterborough | 15852 | 7.90 | 4139 | 2.06 | 19991 | 9.97 |
| NHS Northamptonshire | 15612 | 7.78 | 118 | 0.06 | 15730 | 7.84 |
| NHS North East and North Cumbria | 7596 | 3.79 | 7161.5 | 3.57 | 14757.5 | 7.36 |
| NHS Sussex | 12852 | 6.41 | 1031 | 0.51 | 13883 | 6.92 |
| NHS Humber and North Yorkshire | 8631 | 4.30 | 1812 | 0.90 | 10443 | 5.21 |
| NHS Suffolk and North East Essex | 10284 | 5.13 | 0 | 0.00 | 10284 | 5.13 |
| NHS Devon | 8040 | 4.01 | 0 | 0.00 | 8040 | 4.01 |
| NHS West Yorkshire | 5325 | 2.66 | 2676 | 1.33 | 8001 | 3.99 |
| NHS Buckinghamshire,   Oxfordshire and Berkshire West | 5484 | 2.73 | 142.5 | 0.07 | 5626.5 | 2.81 |
| NHS Lincolnshire | 5508 | 2.75 | 0 | 0.00 | 5508 | 2.75 |
| NHS North East London | 5316 | 2.65 | 0 | 0.00 | 5316 | 2.65 |
| NHS South East London | 4824 | 2.41 | 0 | 0.00 | 4824 | 2.41 |
| NHS Coventry and Warwickshire | 1476 | 0.74 | 2294.5 | 1.14 | 3770.5 | 1.88 |
| NHS Kent and Medway | 2844 | 1.42 | 596 | 0.30 | 3440 | 1.72 |
| NHS Norfolk and Waveney | 3024 | 1.51 | 0 | 0.00 | 3024 | 1.51 |
| NHS Bedfordshire,  Luton and Milton Keynes | 2424 | 1.21 | 75 | 0.04 | 2499 | 1.25 |
| NHS Gloucestershire | 2292 | 1.14 | 0 | 0.00 | 2292 | 1.14 |
| NHS Mid and South Essex | 1620 | 0.81 | 540 | 0.27 | 2160 | 1.08 |
| NHS South Yorkshire | 1812 | 0.90 | 0 | 0.00 | 1812 | 0.90 |
| NHS Surrey Heartlands | 1524 | 0.76 | 228 | 0.11 | 1752 | 0.87 |
| NHS Cheshire and Merseyside | 1404 | 0.70 | 75 | 0.04 | 1479 | 0.74 |
| NHS Hampshire and the Isle of Wight | 600 | 0.30 | 742.5 | 0.37 | 1342.5 | 0.67 |
| NHS Lancashire and South Cumbria | 1224 | 0.61 | 0 | 0.00 | 1224 | 0.61 |
| NHS Derby and Derbyshire | 1092 | 0.54 | 60 | 0.03 | 1152 | 0.57 |
| NHS Staffordshire and Stoke-on-Trent | 0 | 0.00 | 748 | 0.37 | 748 | 0.37 |
| NHS Greater Manchester | 0 | 0.00 | 684.5 | 0.34 | 684.5 | 0.34 |
| NHS North West London | 336 | 0.17 | 345 | 0.17 | 681 | 0.34 |
| NHS Black Country | 636 | 0.32 | 0 | 0.00 | 636 | 0.32 |
| NHS Herefordshire and Worcestershire | 456 | 0.23 | 0 | 0.00 | 456 | 0.23 |
| NHS Hertfordshire and West Essex | 432 | 0.22 | 0 | 0.00 | 432 | 0.22 |
| NHS North Central London Integrated | 312 | 0.16 | 28 | 0.01 | 340 | 0.17 |
| NHS Bath and North East Somerset,   Swindon and Wiltshire | 324 | 0.16 | 0 | 0.00 | 324 | 0.16 |
| NHS South West London | 216 | 0.11 | 30 | 0.01 | 246 | 0.12 |
| NHS Nottingham and Nottinghamshire | 228 | 0.11 | 0 | 0.00 | 228 | 0.11 |
| NHS Bristol, North Somerset  and South Gloucestershire | 96 | 0.05 | 0 | 0.00 | 96 | 0.05 |
| NHS Dorset Integrated | 36 | 0.02 | 0 | 0.00 | 36 | 0.02 |
| NHS Somerset Integrated | 12 | 0.01 | 0 | 0.00 | 12 | 0.01 |
| National Totals | 161172 | 80.37 | 39375.5 | 19.63 | 200547.5 |  |

Supplementary table 4: Data for figure 4 describing the number of DDDs of HCDs issued per geographical UK region in both primary and secondary care across the study period (2019-2022), as well as combined totals.

| Region | Primary |  | Secondary |  |  | Combined |  |  |
| --- | --- | --- | --- | --- | --- | --- | --- | --- |
|  | Sodium Oxybate | Pitolisant | Sodium Oxybate | Pitolisant | Solriamfetol | Sodium Oxybate | Pitolisant | Solriamfetol |
| London | 11004 | 403 | 103140 | 89096 | 1722 | 114144 | 89499 | 1722 |
| North East and Yorkshire | 13896 | 21118 | 35655 | 112292 | 5481 | 49551 | 133410 | 5481 |
| North West | 2628 | 760 | 89364 | 53753 | 1050 | 91992 | 54513 | 1050 |
| South East | 23304 | 2740 | 39828 | 4779 | 434 | 63132 | 7519 | 434 |
| Midlands | 56436 | 19070 | 20162 | 8248 | 1638 | 76598 | 27318 | 1638 |
| South West | 10800 | 0 | 6433 | 2356 | 346 | 17233 | 2356 | 346 |
| East of England | 33636 | 4754 | 5256 | 1107 | 140 | 38892 | 5861 | 140 |

Supplementary table 5(a) + (b): Summary tables of total volumes of DDDs issued per geographical region, showing volumes issued in the starting year of the data set (2019) and the last year of the dataset (2022). Additionally, average volumes issued per month per region across these time intervals.

| (a) Region | Sodium Oxybate DDDs |  | % Change | Mean DDDs/month |  |  |  |
| --- | --- | --- | --- | --- | --- | --- | --- |
|  | **2019** | **2022** |  | **2019** | (SD) | **2022** | (SD) |
| **East of England** | 16464 | 18168 | 10.35 | 1372 | 263.71 | 1514 | 216.23 |
| **London** | 26208 | 36732 | 40.16 | 2184 | 401.24 | 3061 | 483.27 |
| **Midlands** | 30734.40 | 33180 | 7.96 | 2561.20 | 337.89 | 2765 | 331.74 |
| **North East and Yorkshire** | 15219.07 | 16680 | 9.60 | 1268.26 | 264.22 | 1390 | 240.75 |
| **North West** | 22716 | 26448 | 16.43 | 1893 | 326.68 | 2204 | 472.43 |
| **South East** | 17052 | 24396 | 43.07 | 1421 | 301.43 | 2033 | 263.82 |
| **South West** | 5136 | 8065.33 | 57.04 | 428 | 90.28 | 672 | 154.13 |

| (b) Region | Pitolisant DDDs |  | % Change | Mean DDDs/month |  |  |  |
| --- | --- | --- | --- | --- | --- | --- | --- |
|  | **2019** | **2022** |  | **2019** | (SD) | **2022** | (SD) |
| **East of England** | 1402.25 | 884.5 | -36.92 | 116.85 | 45.52 | 73.71 | 41.71 |
| **London** | 16605 | 33060 | 99.10 | 1383.75 | 527.55 | 2755 | 737.21 |
| **Midlands** | 4257.5 | 9433.5 | 121.57 | 354.79 | 171.29 | 786.13 | 150.21 |
| **North East and Yorkshire** | 31369.5 | 34901.5 | 11.26 | 2614.13 | 511.19 | 2908.46 | 915.74 |
| **North West** | 4575 | 22236.5 | 386.04 | 415.91 | 203.59 | 1853.04 | 703.42 |
| **South East** | 885 | 1146.5 | 29.55 | 98.33 | 121.83 | 163.78 | 190.22 |
| **South West** | 194 | 1080 | 456.70 | 97 | 94.75 | 216 | 133.15 |

**Data S1**

Definitions:

- English Prescribing Dataset (‘EPD’): Database of primary care prescriptions collated from data from all primary care practices in NHS England. Provides data on the number, volume, and cost of prescriptions issued in Primary care.
- Secondary Care Medicines Database (‘SCMD’): Database of processed pharmacy stock data, collected from all NHS secondary care trusts in England. Provides a report on the volumes of medications (and some non-pharmaceutical treatments) handled by pharmacies in secondary trusts (including Mental Health and Community Trusts).
- SNOMED CT: An internationally recognised structured collection of clinical terms used to categorise all medical problems, treatments, procedures, and other medical concepts to facilitate the documentation of day-to-day care. It is designed in such a way that differing terminologies are clustered together to enable fluent reporting of unified concepts. In the instance of medications – this means that equivalent drugs and formulations can be identified under one unifying concept (e.g. the active ingredient)
- NHS Dictionary of Medicines and  Devices (dm+D):  A UK based ‘dictionary’ for representing medicines and devices used in the NHS. Each treatment can be subdivided into 5 separate classes, to help pharmacies & hospitals categorise and catalogue available stock. Each medicine or device listed has an associated SNOMED CT code. (<https://dmd-browser.nhsbsa.nhs.uk/>)
- Virtual Medicinal Product: A subclass of the NHS dm+D which clusters clinically equivalent actual medicinal products. For example “Paracetamol 500mg tablets” - which would include all different brands of paracetamol 500mg. Medications in the SCMD are reported as their Virtual Medicinal Product^31^
- Defined Daily Dose: A WHO defined measure – equivalent to the assumed daily maintenance dose for an average adult using a drug for it’s primary indication. (<https://www.who.int/tools/atc-ddd-toolkit/about-ddd>)
- Integrated Care Board: 42 Statutory bodies responsible for planning/funding most local NHS services, covering roughly equal populations across NHS England.

Key Data Sources:

- Primary care prescription data was accessed from the English Prescribing Dataset, provided open-source by NHS-BSA (<https://opendata.nhsbsa.net/dataset/english-prescribing-data-epd>)
- Secondary Care pharmacy stock data was accessed from Secondary Care Medicines Database, provided open source by NHS-BSA - (<https://opendata.nhsbsa.net/dataset/secondary-care-medicines-data>)
- Pharmacy stock codes and meta-data related to each drug and their formulations was attained from the NHS Dictionary of Medicines and Devices (‘dm+d’) – (<https://www.nhsbsa.nhs.uk/pharmacies-gp-practices-and-appliance-contractors/dictionary-medicines-and-devices-dmd>)
- Geographical data on trust, STP, and region locations were attained from a combination of two resources: NHS Digital ‘GP Mapping’ file, providing STP and region names mapped to STP and regions ODS codes (<https://digital.nhs.uk/data-and-information/publications/statistical/patients-registered-at-a-gp-practice/september-2020>) and NHS Digital ‘etr’ which maps trust organisation codes to trust names, STP ODS codes and region ODS codes (<https://digital.nhs.uk/services/organisation-data-service/export-data-files/csv-downloads/other-nhs-organisations>)

Codelist:

| type | id | bnf_code | nm | ingredient | ddd |
| --- | --- | --- | --- | --- | --- |
| vmp | 3.89E+16 | NA | Solriamfetol 75mg tablets | Solriamfetol hydrochloride | 150 |
| vmp | 3.89E+16 | NA | Solriamfetol 150mg tablets | Solriamfetol hydrochloride | 150 |
| vmp | 3.35E+16 | 0404000W0AAAAAA | Pitolisant 18mg tablets | Pitolisant hydrochloride | 18 |
| vmp | 3.35E+16 | 0404000W0AAABAB | Pitolisant 4.5mg tablets | Pitolisant hydrochloride | 18 |
| vmp | 3.96E+08 | 0401010ACAAAAAA | Sodium oxybate 500mg/ml oral solution sugar free | Sodium oxybate | 7.5 |
